# Adolescents’ Developing Sensitivity to Orthographic and Semantic Cues During Visual Search for Words

**DOI:** 10.3389/fpsyg.2019.00642

**Published:** 2019-03-26

**Authors:** Nicolas Vibert, Jason L. G. Braasch, Daniel Darles, Anna Potocki, Christine Ros, Nematollah Jaafari, Jean-François Rouet

**Affiliations:** ^1^Centre de Recherches sur la Cognition et l’Apprentissage, CNRS UMR 7295, Université de Poitiers, Université François Rabelais de Tours, Maison des Sciences de l’Homme et de la Société, Poitiers, France; ^2^Department of Psychology, The University of Memphis, Memphis, TN, United States; ^3^Unité de Recherche Clinique Pierre Deniker du Centre Hospitalier Henri Laborit, INSERM CIC-P 1402, INSERM U 1084 Laboratoire de Neurosciences Expérimentales et Cliniques, CHU de Poitiers, Groupement De Recherche CNRS 3557, Université de Poitiers, Poitiers, France

**Keywords:** visual search, word search, reading proficiency, lexical quality, eye-tracking, development, children, adolescents

## Abstract

Two eye-tracking experiments were conducted to assess the influence of words either looking like the target word (orthographic distractors) or semantically related to the target word (semantic distractors) on visual search for words within lists by adolescents of 11, 13, and 15 years of age. In Experiment 1 (literal search task), participants saw the target word before the search (e.g., “raven”), whereas in Experiment 2 (categorical task) the target word was only defined by its semantic category (e.g., “bird”). In both experiments, participants’ search times decreased from fifth to ninth grade, both because older adolescents gazed less often at non-target words during the search and because they could reject non-target words more quickly once they were fixated. Progress in visual search efficiency was associated with a large increase in word identification skills, which were a strong determinant of average gaze durations and search times for the categorical task, but much less for the literal task. In the literal task, the presence of orthographic or semantic distractors in the list increased search times for all age groups. In the categorical task, the impact of semantic distractor words was stronger than in the literal task because participants needed to gaze at the semantic distractors longer than at the other words before rejecting them. Altogether, the data support the assumption that the progressive automation of word decoding up until the age of 12 and the better quality of older adolescents’ lexical representations facilitate a flexible use of both the perceptual and semantic features of words for top-down guidance within the displays. In particular, older adolescents were better prepared to aim at or reject words without gazing at them directly. Finally, the overall similar progression of the maturation of single word visual search processes and that of more real-life information search within complex verbal documents suggests that the young adolescents’ difficulties in searching the Web effectively could be due to their insufficiently developed lexical representations and word decoding abilities.

## Introduction

In daily life, inquirers of all ages often visually search through verbal displays (e.g., lists, continuous texts) to locate pieces of information such as the name of objects, places, or people. A wealth of empirical research has examined adults’ visual search for non-verbal items such as shapes or symbols (for reviews, Thornton and Gilden, 2007; Zelinsky, 2008; Wolfe, 2014; Wolfe and Horovitz, 2017). However, research on the visual search for verbal information such as words or simple phrases is still scarce (for reviews, Dampuré et al., 2012, 2014; Léger et al., 2012; Boettcher and Wolfe, 2015; Zhou et al., 2017).

Previous research demonstrated that non-verbal visual search proficiency progressively increases during childhood and adolescence (for reviews, Trick and Enns, 1998; Hommel et al., 2004; Donnelly et al., 2007; Burggraaf et al., 2018). Children and teenagers search faster as age increases, and eye-tracking data reveal that this mainly results from a decrease of the average duration of the fixations made on the items in the search display (Huurneman and Boonstra, 2015; Burggraaf et al., 2018). However, very few studies focused on the development of search for lexical targets among lexical information (Leslie and Calfee, 1971; Lefton and Fisher, 1976; Bisanz and Resnick, 1978; Stanovich and West, 1978, 1979; Stanovich et al., 1978; Seassau and Bucci, 2013). In addition, the data from these studies are somewhat limited, because they did not use any online measurement technique such as eye-tracking.

The present work argues that the examination of adolescents’ proficiency in visual search for words becomes a pressing issue. Indeed, understanding how children and teenagers visually scan verbal displays is essential in explaining some of the challenges they face when using complex information systems such as search engines, social networking sites, or web pages. Reading from these new types of texts involves a large amount of visual scanning as well as the dismissal of irrelevant information to avoid distraction and feeling lost (Kuiper et al., 2005). Evidence from research studies (Kobasigawa, 1983; Dreher and Sammons, 1994; Hirsh, 2000; Rouet and Coutelet, 2008; Kaakinen et al., 2015; Potocki et al., 2017) and large-scale assessments (e.g., Organisation for Economic Co-operation and Development [OECD], 2011) suggest that these abilities develop gradually throughout both childhood and adolescence. Hence, the current research focused on the development of the basic component processes involved in visual search for words by looking at the way adolescents explore word lists when searching for a target word. Models of non-lexical visual search were used as a framework to investigate how the visual and semantic properties of words interact to guide adolescents’ attention within the list and influence search performance. The presence within the lists of various kinds of potentially distracting words was manipulated. Accordingly, the experiments were conducted with three goals in mind: to document the development of visual search for words throughout adolescence, to elucidate whether the patterns of development differ depending on what the searcher knows about the target word, and to relate the findings to the optimization of adolescents’ visual search in Internet-like environments.

### Visual Search for Words by Adults

Visual search models (Thornton and Gilden, 2007; Wolfe, 2014) posit that searching involves two stages. First, visual information is processed in parallel over the whole display. If the target differs from distractors by a single visual feature, it “pops-out” immediately in a bottom-up fashion. If not, people engage in a serial process whereby their attention and gaze moves from item to item until the target is found (Zelinsky, 2008). Both bottom-up and top-down mechanisms guide this exploration. Bottom-up guidance is based on the visual saliency of stimuli, whereas top-down guidance depends on the user knowledge and on task requirements (Thornton and Gilden, 2007; Wolfe, 2014; Wolfe and Horovitz, 2017).

During visual search for words, there is no pop-out unless the target word is written in a distinctive font or color (Thornton and Gilden, 2007; Dampuré et al., 2014). Hence, when no such cue is available, the searcher must actively scan the display to find the target word (Thornton and Gilden, 2007). When the searcher fixates a distractor word, she or he must reject it as quickly as possible using foveal vision, but must also decide where to look next using what is perceived of the other words present in the parafoveal/peripheral visual field. According to models of visual search, the item that has the most features in common with the representation of the target (the target “template”) kept in working memory should attract attention. More precisely, memorizing the target would pre-activate the target’s visual features and guide attention toward items with similar features. Once selected, the attended items enter a limited-capacity recognition process in which distractors are rejected as soon as one of their features differs from that of the target. With words, one can ask whether attention is attracted by visual similarity and/or semantic relatedness with the target word and whether this guidance process is modulated by searchers’ goals.

A few studies addressed these questions in adults who searched for single target words within either word lists (Léger et al., 2012), or random word displays (Dampuré et al., 2014; Zhou et al., 2017). Léger et al. (2012) and Dampuré et al. (2014) manipulated visual similarity between words through orthographic overlap, and semantic relatedness through levels of semantic association between the target word and the other words in the display. The same material was used in two distinct tasks, namely a “literal” task in which participants knew the target word (e.g., “raven”), and a “categorical” task in which the target was only defined by its superordinate category (e.g., “bird”). Eye movement recordings informed about which words were fixated during the search process. The number of fixations made on each word was used to evaluate its ability to attract participants’ attention when perceived in the parafoveal/peripheral visual field, while gaze durations were used to evaluate the amount of processing needed to reject distractor words using foveal vision (Zelinsky, 2008).

As expected, participants’ search times and eye movements varied according to both the nature of the search task and the characteristics of distractor words (Léger et al., 2012; Dampuré et al., 2014). In the literal task, the presence of words looking like the target word (orthographic distractors) strongly increased search times, because they were more often fixated (i.e., they attracted participants’ attention) and fixated for longer durations (i.e., participants needed more time to reject them) than other distractor words. Perceiving words that look like the target template kept in working memory would attract the participants’ attention, and they would be difficult to reject because of their visual similarity with the target word. The inclusion of words related semantically to the target word (semantic distractors) also increased search time because these words could attract participants’ attention. On learning the target word, people would activate the lexical representations of semantically related words. Then, words having a semantic relationship with the target would attract attention during the search phase because their visual form matches that of words that were pre-activated in memory. However, semantic distractors were not fixated for longer durations than other words, probably because they did not resemble the target word and were thus easily rejected.

In the categorical task, Léger et al. (2012) found that the presence of semantic distractors led to target selection errors, but did not increase search times or modify the number or duration of fixations made on distractor words. In contrast, orthographic distractors slowed down search time because even though they no longer attracted participants’ attention, participants needed more time to reject them than to reject neutral words. Hence, orthographic similarity between distractor words and the target influenced visual search even when participants did not known the target word in advance.

### Children and Adolescents’ Visual Search for Verbal Information

The few former studies of visual search for lexical information by children offer insight into potential factors affecting children’s search efficiency. Firstly, the speed and efficiency of visual search for letters or words strongly increases between 7 and 11–12 years of age (Leslie and Calfee, 1971; Bisanz and Resnick, 1978; Stanovich et al., 1978). Whereas efficient letter search is achieved by age 12 (Bisanz and Resnick, 1978), the efficiency of word search goes on increasing into adulthood (Leslie and Calfee, 1971; Stanovich et al., 1978). However, no studies have directly tested the development of visual search for words between the ages of 12 and 18.

Secondly, younger searchers or less skilled readers are supposed to be particularly sensitive to the orthographic characteristics of distractive lexical information when searching for words. Indeed, the level of orthographic similarity between targets and non-targets strongly influences third graders’ visual search performance (Stanovich et al., 1978; Stanovich and West, 1979). Mediated priming paradigms confirmed that third graders are allocating more cognitive resources toward the orthographic features of words than sixth graders and adults (Reimer, 2006). These results are in accordance with Gibson’s early theory of the development of word perception (Gibson, 1971), which stated that while children learn to read and develop fluency in word identification, they become less aware of the orthographic features of words and more spontaneously attentive to word meaning. More recently, this theory was extended in the framework of the lexical quality hypothesis (Perfetti and Hart, 2002), as detailed below.

### The Lexical Quality Hypothesis

With age, readers’ vocabulary increases both quantitatively (they know more words) and qualitatively (they refine their knowledge of word meanings). Indeed, the richness and flexibility of readers’ lexical representations, namely the quality and number of connections among concepts that the word evokes within the reader’s semantic memory, are critical for word use (Beck et al., 1982; Björklund, 1987; Perfetti and Stafura, 2014). More precisely, the lexical quality hypothesis states (Perfetti, 2007; Verhoeven and Perfetti, 2011; Verhoeven et al., 2011) that the mental representations of words include three main features, i.e., their orthographic, phonological, and semantic properties. Another feature – the constituent binding – refers to the degree to which these three features are connected together. High quality lexical representations include well-specified orthographic, phonological and semantic features that are strongly connected to one another.

Classical models of reading assume that expert readers retrieve all constituents synchronously during word recognition (Coltheart et al., 2001). However, according to the lexical quality hypothesis, this would not be the case in younger or less skilled readers (Perfetti and Hart, 2002; Nation, 2009). Indeed, the phonological and orthographic representations of words are established first, whereas their integration with meaning comes henceforth (Vellutino et al., 2007; Richter et al., 2013). As children get older, they progressively acquire higher quality lexical representations that enable them to retrieve rapidly the meaning of words (Perfetti, 2007; Verhoeven and Perfetti, 2011).

According to models of the acquisition of word recognition skills (Stanovich, 1980; Perfetti, 1992; Plaut and Booth, 2000), decoding of the word’s visual form plays a central role in children’s reading and vocabulary development. Hence, the progressive automation of word decoding and attainment of a fluent level of reading between first and sixth grades would also free mental resources for semantic processing and closer consideration of word or text meaning (Laberge and Samuels, 1974; Vellutino et al., 1981). Similarly, the development of the readers’ word decoding abilities might also free up resources for a larger use of the semantic properties of words during visual search. This should give them faster access to the meaning of words during the search tasks, but should also increase the likelihood that they would be attracted by words that are semantically related to the target word in their visual field.

Adolescents’ developing word decoding skills and quality of lexical representations may influence their behavior in more complex reading tasks involving the scanning and detection of relevant words, e.g., during a web search. Indeed, several studies investigated how children visually search for information on search engine result pages. The data indicate that developing readers, including young adolescents, may be more sensitive to surface features such as typographical emphasis or the presence of particular keywords, than to the deeper meaning of the phrase under examination (e.g., Rouet et al., 2011; Keil and Kominsky, 2013; for reviews, Walraven et al., 2008; Zhang and Quintana, 2012). For instance, using simulated search engine result pages, Rouet et al. (2011) found that 6th graders tended to select irrelevant phrases (e.g., the highest train in the world) in response to a search phrase (e.g., the highest mountains in the world) if the matching keywords were capitalized (e.g., the HIGHEST train in the WORLD).

Altogether, the literature suggests that the impact of semantic distractors during visual search for words should increase with age in relation with the automation of word decoding and acquisition of higher quality lexical representations. Since typical readers are considered to reach adult-like fluency in word decoding by the sixth grade, i.e., at about 12 years of age (Vellutino et al., 1981; Björklund, 1987; Verhoeven and van Leeuwe, 2008; Verhoeven et al., 2011), the present research investigated the development of visual search for words in 10–16 years old fifth, seventh, and ninth graders.

### The Current Study

The current research sought to investigate the factors that influence adolescents’ visual search for words, and to document the degree to which the search process develops between 10 and 16 years of age. Fifth, seventh, and ninth graders were provided with a target word (Experiment 1), or with a categorical cue about the target word (Experiment 2). They visually searched for the target through lists of words, which could include potentially distracting words that were either orthographically similar (“orthographic distractors”) or semantically related (“semantic distractors”) to the target word. Participants’ eye movements were recorded to determine which words were fixated and for how long. As in previous developmental visual search studies, the speed of visual search for words was expected to increase with age in both experiments, mainly because of a decrease of the average duration of fixations made on distractor words (Burggraaf et al., 2018).

In Experiment 1 (“literal” search), orthographic distractors were expected to increase adolescents’ search times just as they do in adults (Léger et al., 2012; Dampuré et al., 2014). Semantic distractors were also expected to increase search times (Léger et al., 2012), and the impact of semantic distractors was expected to *increase* with age, due to a more automatic activation of words’ semantic features during search (Perfetti, 2007; Verhoeven and Perfetti, 2011).

In Experiment 2 (“categorical” search), no significant variation of the number of gazes made on non-target words was expected according to the type of distractors included in the list. Indeed, data from adults (Léger et al., 2012) suggests that in the situation of this experiment, words’ orthographic or semantic features cannot be used for top-down attention guidance. The impact of orthographic distractors on gaze durations and search times seen in adults was expected to *increase* with age because it involves a pre-activation of potential target words that is mediated by semantic features, i.e., by automatic spreading activation within the semantic network activated upon seeing the target word category name (see Léger et al., 2012 for more details). As such, the impact of orthographic distractors in the categorical task should increase with age just like the impact of semantic distractors in the literal task. Semantic distractors were expected to have a stronger impact on adolescents’ gaze durations and search times compared to both adults and to the literal task. Indeed, because the target word is not known in advance, the searchers must read the words to assess their meaning (see Léger et al., 2012), and must actively inhibit the semantic distractors. Since both vocabulary knowledge and active inhibition abilities mature between 9 and 16 years of age (Brocki and Bohlin, 2004), the semantic distractors should be more disruptive for adolescents than for adults.

To investigate the links between reading fluency and visual search for words, participants’ reading proficiency was assessed using a word identification test (Lefavrais, 1968). Participants’ scores were expected to strongly increase between fifth and seventh grades, but much less afterward. In Experiment 1, only a weak relationship was expected between adolescents’ reading proficiency and search times (Leslie and Calfee, 1971; Stanovich et al., 1978), because participants do not need to access the lexical representation of non-target words to reject them. In Experiment 2, a stronger, positive relation was expected between participants’ search efficiency and reading proficiency because the meaning of non-target words must be accessed.

In the rest of this paper, we first describe the methods of both experiments, before presenting the results and discussions of Experiments 1 and 2 in two separate sections. What the data reveal about the development of visual search for words and the practical implications of the data are discussed in the general discussion section.

## Materials and Methods of Experiments 1 and 2

### Participants

The experiments were conducted in four schools nearby a middle-sized French city. Fifth graders were recruited in two elementary schools, while seventh and ninth graders were recruited from two junior high schools. Students came from above average socio-economic backgrounds in one of the elementary schools and one of the junior high schools, and from low or mixed socio-economic backgrounds in the two others. Written and informed permission was sought from the school, the teacher, and from all students’ parents or legal guardians by sending a parental consent form. After permission was obtained, all students were read a verbal script, either individually or during a whole-class session, and were asked to participate in the study. All children who accepted to participate were included. Full review and approval of the study by an ethics committee or an Institutional Review Board was not required according to institutional and national guidelines and regulations.

The same 114 students participated in both experiments. All had normal or corrected-to-normal vision and were native French speakers. The sample included 42 fifth graders (*M* age = 10.7 years, *SD* = 0.4, range 10.2–11.6 years, 18 females), 36 seventh graders (*M* age = 12.9 years, *SD* = 0.5, range 12.2–14.2 years, 16 females), and an older group of 36 students including 32 ninth graders, 3 tenth graders, and 1 eleventh grader (*M* age = 15.0 years, *SD* = 0.7, range 14.0–16.4 years, 19 females). This last group will be named the “ninth grade” group for sake of simplicity.

### Apparatus

Participants’ eye movements were recorded using a TOBII 1750 eye-tracker. The stimuli were displayed on a 17-inch monitor with a resolution of 1,024 × 768 pixels. A Fujitsu/Siemens laptop controlled the eye-tracker, which provided gaze positions at a sampling frequency of 50 Hz.

The distance between participants’ eyes and the eye-tracker was adjusted to get the best eye detection and eye movement recordings as possible. Depending on the participants, the optimal viewing distance varied between 493 and 757 mm (*M* = 595 mm, *SD* = 55). Léger et al. (2012) estimated the effective precision of the eye-tracker at *M* = 0.54° of visual angle (*SD* = 0.30), which in the present experiment corresponded to 4.6–7.1 mm on the screen depending on the viewing distance.

### Visual Search Material

The visual search material for Experiments 1 and 2 consisted of 84 lists of French nouns. There were nine words (a target word and eight other words) in each list. The words were displayed left-aligned in a single column in the center of the screen, and were written in lower case letters in size 14 Verdana font. Each word appeared 6 mm high on the screen of the eye-tracker (i.e., 0.45–0.69° of visual angle depending on the viewing distance), and there was a 21 mm wide space between the lower limit of a word and the upper limit of the following word in the list (i.e., 1.59–2.41° of visual angle). Hence, there was at least 2.04° of visual angle vertical spacing between successive words’ midpoints, which was almost four times larger than the precision of the eye-tracker.

All 552 words used in the lists had 4 to 10 letters (*M* = 7.1, *SD* = 1.3) and 2 or 3 syllables. Most of them (547 out of 552, 99.1%) had a lexical frequency greater than two per million in corpora of third- to fifth-grade readers used in French elementary schools, according to the Manulex (Lété et al., 2004) and/or Novlex (Lambert and Chesnet, 2001) databases. The lexical frequency of the five last words varied between 0.5 and 1.5 per million.

The 84 lists included 12 filler lists and 24 sets of three experimental lists. Each set of experimental lists was built around a target word that was an exemplar from a category, which was used in Experiment 2 to define the target word (Table 1). For example, “corbeau” (raven) was an instance of “oiseau” (the bird category).

**Table 1:** List of the 24 categories and target words used for the experimental lists.

Target word	Category	Target word	Category	Target word	Category
corbeau (raven)	oiseau (bird)	bassine (basin)	récipient (container)	couteau (knife)	ustensile (utensil)
anémone (anemone)	fleur (flower)	poumon (lung)	organe (organ)	salon (lounge)	pièce (room)
chemise (shirt)	vêtement (clothing)	essence (petrol, gas)	liquide (liquid)	mouton (sheep)	animal (animal)
salade (salad)	légume (vegetable)	camion (truck, lorry)	véhicule (vehicle)	acier (steel)	métal (metal)
serpent (snake)	reptile (reptile)	abeille (bee)	insecte (insect)	mandarine (mandarin)	fruit (fruit)
armoire (wardrobe)	meuble (piece of furniture)	poupée (doll)	jouet (toy)	sculpteur (sculptor)	artiste (artist)
perceuse (drill)	outil (tool)	requin (shark)	poisson (fish)	pétrolier (tanker)	bateau (boat)
champagne (champagne)	boisson (drink)	poignard (dagger)	arme (weapon)	baignade (bathing)	loisir (leisure)

*The French target words and category names are written in the Verdana font seen by participants.*

In most cases (15 out of 24), the target word was one of the ten most frequently produced exemplars from its category (Tourrette, 1979; Léger et al., 2008). Five target words were less typical exemplars from their category, whereas no data were found for the four last words. According to the Manulex database, the average lexical frequency of the 24 experimental target words was 35.7 per million (range 1.5–117.9). The three different experimental word lists were built around each of the 24 target words (see Table 2 for an example) as follows:

**Table 2:** Experimental lists for the target word “corbeau” (raven), which was used as an exemplar of “oiseau” (bird).

	Orthographic list		Semantic list		Neutral list	
	French words	Translation	French words	Translation	French words	Translation
Target word	corbeau	raven	corbeau	raven	corbeau	raven
Distractor words	cadeau	present	branchage	branches	action	action
	carreau	tile, pane	forêt	forest	grimace	grin
	chameau	camel	perchoir	perch	liaison	link
	ciseau	scissors	plumage	plumage	paupière	eyelid
Filler words	basket	basket-ball	basket	basket-ball	basket	basket-ball
	moyenne	average	moyenne	average	moyenne	average
	roulette	roulette, caster	roulette	roulette, caster	roulette	roulette, caster
	serrure	lock	serrure	lock	serrure	lock

*The French versions of the words are written as they appeared to the participants.*

– The first type of list (“orthographic list”) included the target word, four orthographically similar distractor words that shared the first and last two letters with the target word, and four neutral “filler” words that were unrelated to the target word.
– The second type of list (“semantic list”) included the target word, four distractor words, which were semantically related to both the target word and the name of its category, and the same four neutral filler words as in the orthographic list. The semantic distractors were *not* other instances of the category. The semantic association levels between the target word, the name of its category and each semantic distractor were rated in a previous experiment by 70 adult volunteers who judged the level of semantic association between the different pairs of words (Léger et al., 2012). All words chosen as semantically related distractors were perceived as having a significant semantic association with both the target words and their categories.
– The third type of list (“neutral list”) included the same filler words as in the previous lists and four additional neutral words (“neutral distractors”) that were unrelated to the target word.

The position in the list of the target word and four filler words was held constant across the three experimental lists. The positions of the four list-specific distractor words were also the same in all lists, but their nature (orthographic, semantic, or neutral) changed according to list type (Table 2).

Previous visual search studies using eye movement recordings demonstrated that the placement of words in a central column within search displays led most participants to scan them from top to bottom (Ojanpää et al., 2002; Léger et al., 2006). As such, the target word was always placed within the bottom half of the lists for the experimental trials (positions 5 through 9). The position of the target word was randomized to one of the five possible slots so that the distribution of target words over the 24 trials was not significantly different from a homogeneous distribution [χ^2^(4) = 2.33, *p* = 0.67].

For the 12 filler trials, in contrast, the target word was always placed within the top half of the list (positions 1 to 4) to prevent the participants from focusing on the end of the lists. Once the location of the target word was set, the non-target words were randomly distributed across the remaining positions. The target words of the 12 filler lists (which included four orthographic, four semantic and four neutral lists) differed from those of experimental lists. None of the words included in the experimental or filler lists were used more than once.

### Word Identification Speed

The participants’ word identification speed was measured using the “Pipe and Rat” test (Lefavrais, 1968). Participants had to read silently three pages of single words displayed on successive lines with articles (beginning with “the pipe – the rat – the motorbike – the louse – the mule –...”). There were on average six words per line. They had to read as many words as possible within 3 min. Half of the words were animal names and the participants were tasked to underline all animal names while reading. The number of correctly identified animal names minus the number of incorrectly underlined names (maximum 283) was an index of word identification speed.

### Design and Procedure

Participants were individually tested. Half of them began with Experiment 1, the other half with Experiment 2. In Experiment 1 (“literal” search task), the target word (e.g., raven) was shown to participants before each trial. In Experiment 2 (“categorical” search task), the participants did not know the word they had to find, but saw the name of a category (e.g., bird) and had to search within the list for the single instance of this category (raven).

In each experiment, following eye movement calibration, participants were presented with three practice trials, followed by a series of 12 experimental and six filler trials in random order. The target words used in the practice trials were included in neutral lists, and differed from the experimental and filler target words. The assignment of the 24 target words between the three types of lists and the two experiments was counterbalanced so that in each experiment, the type of list was crossed with four groups of six participants and three sets of target words (four words per set). The same practice and filler trials were used for all participants.

Each trial began with a slide where the words “essai suivant” (next trial) were shown together with a cross that was located in the middle of the left side of the screen, on which the participant had to place the mouse cursor. Then, the experimenter pressed the space bar. As a result, the target word (Experiment 1) or the category name (Experiment 2) appeared at the screen top left corner. The experimenter read the word aloud, and then pressed the space bar again. This advanced to a screen displaying the list in which the participant was to locate the target word. Once the target word was located, the participant had to click on it. Then the experimenter pressed the space bar again, cycling through the same procedure for subsequent trials. Participants had to find the target word as fast as possible and to click on it as soon as they had found it. The response time was the time that elapsed between the word list appearance and the participant’s click on the target word.

To control for age- and computer experience-related differences in mouse control between children, a short, speeded mouse-pointing task was performed twice by each participant: once prior to beginning the experiments and once at the end of the session. The task included five trials that began with the same slide as the visual search trials. Once the participant had positioned the mouse cursor on the cross, the experimenter pressed the space bar. This advanced to a screen displaying an 18 mm width and 76 mm length rectangle, which was located at one of the nine locations where words were displayed in the lists. When the rectangle appeared, the participant had to move the mouse and click anywhere within it as quickly as possible. The size of the rectangle was the same as that of the area of interest that was drawn around each word to assign fixations to particular words in the visual search trials (see data analysis below). The location of the rectangle was different on each trial, and the five locations differed between the two instances of the task. For each trial, the “motor reaction time” was defined as the time that elapsed between the rectangle appearance and the moment the participant clicked inside it.

The test measuring word identification speed was administered after the two visual search experiments for seventh and ninth graders, or separately in full-class contexts for fifth graders.

### Eye Movement Recordings

The eye movements and mouse clicks were recorded and analyzed using the ClearView 2.7.1 software. Any period where gaze stopped for 60 ms (i.e., three successive gaze points sampled at 50 Hz) or more within a 30 pixels (0.77 to 1.17° of visual angle depending on the participant) diameter area was defined as an eye fixation.

### Data Analyses

Adolescents’ visual search performance was assessed using error rates, search times and eye movement-related indicators as dependent variables. All trials where participants made errors and failed to click on the target word were excluded from analyses of search times and eye movements. Because of the low error rates and because the data were not normally distributed, non-parametric tests were used to analyze whether error rates were differentially related to grade (fifth, seventh, or ninth) and/or type of list (orthographic, semantic, or neutral).

Linear mixed models were used to analyze search times and eye movement data. In contrast with standard analyses, linear mixed models evaluate the impact of both the independent variables that are manipulated (fixed factors) and the random effects linked to inter-participant and inter-item variability (Kliegl et al., 2010). More precisely, they include random intercepts that account for the variability across participants or items, and random slopes that account for the variability in participants’ or items’ sensitivity to the independent variables. In this study, all linear mixed models were obtained using the “lmer” function of the “lme4” R statistical package (Copyright M. P. Wand 1997–2009) implemented in “R” software (version 3.0.2). As advocated by Barr et al. (2013), each analysis started with the maximal random effects structure and included participants and items as random intercepts, and as many as possible by-participant and by-item random slopes. The random effects that caused the model to fail to converge were then removed, and the model was run again. All the models reported hereafter correspond to this final analysis. The *F*-values and probabilities were obtained using Satterthwaite approximation for degrees of freedom (Satterthwaite, 1946; Keselman et al., 1999). All follow-up pairwise comparisons were performed based on adjusted least-squares means.

To control for differences in mouse control ability, the median “motor reaction time” obtained by each child on the two speeded mouse-pointing tasks was subtracted from their response time to isolate their “search time.” The search times underwent logarithmic transformation, and were then analyzed using linear mixed models with grade (fifth, seventh, or ninth), type of list (neutral, semantic, or orthographic) and the grade by type of list interaction as fixed factors, and participants and items as random intercepts. The fifth grade and the neutral list were taken as references for the fixed effects. As detailed above, the model also included by-participant and by-item random slopes to account for the variable sensitivity of participants and/or items to the effects of the grade and type of list.

The eye movements made during visual search were analyzed using two dependent measures: the number and average duration (in ms) of gazes made on the non-target words before the participant’s click on the target word. A fixation was assigned to a word if it fell within an 18 mm high and 76 mm long rectangle centered on each word. Since each word was 6 mm high and the space between successive words was 21 mm (see above), there was a 9 mm vertical space between the rectangles drawn around each successive word. The fixations that landed within that space were not assigned to a word and were, thus, not taken into account during analysis. Successive fixations on the same word were fused together and were counted as one “gaze” on this word. In contrast, re-fixations on the same word, after at least one fixation elsewhere in the display, were counted as a second gaze on the word.

Logarithmic transformations were applied to the number and average duration of gazes made on non-target words before analyses. To reduce interference due to the hand-eye coordination process that takes place when moving the mouse to click on the target word, the gazes that coincided with the mouse click were excluded. All analyses of eye movements were conducted using linear mixed models with grade, type of list, type of word (filler words or distractor words) and their interactions as fixed factors, and participants and items as random intercepts. The fifth grade, the neutral list and the filler words were chosen as references for the fixed effects. As detailed above, the models included by-participant and by-item random slopes to account for the variable sensitivity of participants and/or items to the effects of the grade, the type of list and the type of words. In particular, as recommended by Barr (2013), the highest-order interaction between within-participant fixed factors, i.e., the type of list by type of word interaction, was always included as a by-participant and by-item random slope in the initial models. However, the inclusion of this interaction as a random slope systematically caused the models to fail to converge, which explains why these random slopes do not appear in the final models used for the analyses.

When the models for search times or eye movement measures revealed a significant main effect of one or both of the three-level factors (i.e., grade and/or type of list), but no significant interaction between them or with the type of word, follow-up pairwise comparisons between grades and/or types of list were performed using dummy contrast coding. The two levels to be compared were set to -1 and 1, respectively, while the remaining level was set to 0.

Analyses of the participants’ word identification scores were first conducted using a one-way ANOVA with grade as a between-participants factor. Because the score depended on grade (see below), its impact on the participants’ search times and eye movements had to be assessed separately for fifth, seventh, and ninth graders and for the literal and the categorical tasks using linear mixed models. The models of the adolescents’ search times included the word identification score and the type of list as fixed factors, and participants and items as random intercepts. The models of the adolescents’ number and duration of gazes made on non-target words included the type of word and the type of word by type of list interaction as additional fixed factors. All models also included by-participant and by-item random slopes as detailed above.

## Literal Search Task (Experiment 1)

### Results

#### Error Rates

In the literal task, participants made only four target selection errors over 1,368 experimental trials, i.e., a 0.3% error rate.

#### Search Times

The upper panel of Table 3 displays the participants’ average visual search time as a function of grade and type of list. The model for search times^1^ revealed main effects for grade [*F*(2,104) = 20.62, *p* < 0.001] and type of list [*F*(2,26) = 12.12, *p* < 0.001] with no significant interaction [*F*(4,1,182) = 0.45, *p* = 0.77]. As expected, the speed of visual search for words increased with age since, regardless of the type of list, follow-up pairwise comparisons revealed that fifth graders’ search times were longer than seventh graders’ [β = -0.073, *SE* = 0.017, *F*(1,107) = 17.86, *p* < 0.001] and ninth graders’ [β = -0.101, *SE* = 0.016, *F*(1,106) = 38.97, *p* < 0.001]. The ninth graders were, however, not significantly faster than the seventh graders [β = -0.030, *SE* = 0.019, *F*(1,106) = 2.31, *p* = 0.13]. As expected also, the presence of either orthographic or semantic distractors reduced search efficiency compared to instances where they were not present. Indeed, search times were longer for both orthographic lists [β = 0.054, *SE* = 0.010, *F*(1,26) = 21.42, *p* < 0.001] and semantic lists [β = 0.028, *SE* = 0.009, *F*(1,42) = 4.52, *p* < 0.05] than for neutral lists. In addition, search times were marginally longer for orthographic lists than for semantic lists [β = 0.022, *SE* = 0.012, *F*(1,22) = 3.49, *p* = 0.08], which suggests that orthographic distractors reduced search efficiency more than semantic distractors.

**Table 3:** Average visual search times (in ms) as a function of grade and type of list in Experiments 1 (literal search) and 2 (categorical search).

	Type of list		
	Neutral	Orthographic	Semantic
**Experiment 1**			
Fifth grade	1,964 (856)	2,552 (869)	1,944 (528)
Seventh grade	1,399 (478)	2,006 (696)	1,586 (623)
Ninth grade	1,240 (417)	1,642 (598)	1,414 (476)
**Experiment 2**			
Fifth grade	4,441 (1,952)	4,364 (1,956)	4,820 (2,637)
Seventh grade	3,501 (1,701)	3,675 (1,195)	3,351 (1,152)
Ninth grade	3,350 (1,684)	3,292 (1,714)	2,994 (1,135)

*The standard deviations computed by participants are provided within parentheses.*

#### Eye Movement Data: Number of Gazes

**FIGURE 1:**
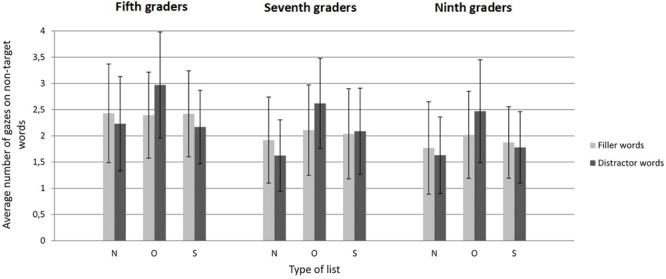
Average number of gazes as a function of grade, type of list, and type of word in Experiment 1 (literal search). Error bars represent standard deviations. N, Neutral; O, Orthographic; S, Semantic.

Figure 1 displays the number of gazes made by participants on non-target words as a function of grade, type of list, and type of word. Adolescents gazed at about half of the non-target words in each list, and stopped searching as soon as they fixated the target word.

The model for the number of gazes^2^ revealed main effects for grade [*F*(2,110) = 8.73, *p* < 0.001] and type of list [*F*(2,20) = 8.83, *p* < 0.01], and an interaction between type of list and type of word [*F*(2,2.499) = 12.33, *p* < 0.001]. The remaining interactions were not significant (all *F*s < 0.66, all *p*s > 0.62). The main effect of grade revealed that part of the increase of search speed with age was due to a decrease of the number of gazes made on non-target words. Indeed, regardless of the type of list and type of word, fifth graders gazed at more non-target words than seventh graders [β = -0.030, *SE* = 0.010, *F*(1,111) = 8.75, *p* < 0.01] and ninth graders [β = -0.040, *SE* = 0.010, *F*(1,110) = 16.36, *p* < 0.001]. In contrast, seventh graders did not gaze at significantly more non-target words than ninth graders [β = -0.011, *SE* = 0.011, *F*(1,111) = 0.95, *p* = 0.33].

The follow-up pairwise comparisons associated with the type of list by type of word interaction demonstrated that regardless of grade, the increase of search times caused by orthographic distractors was due in part to an increase of the number of gazes made on these distractors. Participants gazed more often at orthographic distractors than at neutral distractors [β = 0.116, *SE* = 0.019, *t*(39) = 6.01, *p* < 0.001], whereas the numbers of gazes made on semantic and neutral distractors did not differ significantly [β = 0.026, *SE* = 0.016, *t*(50) = 1.62, *p* = 0.11]. The filler words did not attract significantly different numbers of gazes in orthographic lists [β = 0.021, *SE* = 0.019, *t*(39) = 1.06, *p* = 0.29] and semantic lists [β = 0.008, *SE* = 0.016, *t*(50) = 0.48, *p* = 0.63] compared to neutral lists.

#### Eye Movement Data: Average Gaze Duration

**FIGURE 2:**
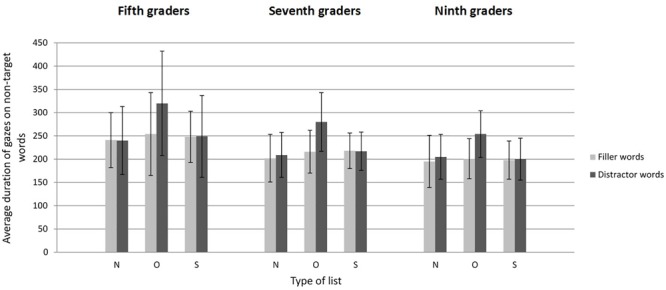
Average duration of gazes as a function of grade, type of list, and type of word in Experiment 1 (literal search). Error bars represent standard deviations. N, Neutral; O, Orthographic; S, Semantic.

Figure 2 displays mean gaze durations on non-target words as a function of grade, type of list, and type of word. The model for the average duration of gazes^3^ revealed main effects for grade [*F*(2, 111) = 13.55, *p* < 0.001], type of list [*F*(2,2.175) = 37.57, *p* < 0.001], and type of word [*F*(1,23) = 25.83, *p* < 0.001]. There was also an interaction between type of list and type of word [*F*(2,2.170) = 33.41, *p* < 0.001]. The remaining interactions were not significant (all *F*s < 1.04, all *p*s > 0.38). The main effect of grade revealed that the increase of search speed with age was not only due to a decrease of the number of gazes made on non-target words, but also to a decrease of the duration of these gazes. Average gaze durations were longer for fifth graders than for seventh graders [β = -0.032, *SE* = 0.009, *F*(1,110) = 11.56, *p* < 0.001] and ninth graders [β = -0.041, *SE* = 0.009, *F*(1,111) = 20.33, *p* < 0.001], but were not significantly different between seventh and ninth graders [β = -0.010, *SE* = 0.010, *F*(1,112) = 0.90, *p* = 0.34].

The follow-up pairwise comparisons associated with the type of list by type of word interaction demonstrated that regardless of grade, the increase of search times caused by orthographic distractors did not only result from an increase of the number of gazes made on non-target words, but also from the longer duration of these gazes. Indeed, adolescents gazed much longer at orthographic distractors than at neutral distractors [β = 0.111, *SE* = 0.011, *t*(2,178) = 10.27, *p* < 0.001], whereas the duration of gazes made on semantic and neutral distractors did not differ significantly [β = 0.004, *SE* = 0.011, *t*(2,180) = 0.39, *p* = 0.69]. Participants displayed similar gaze durations on filler words in orthographic lists [β = 0.012, *SE* = 0.011, *t*(2,180) = 1.11, *p* = 0.27] and semantic lists [β = 0.017, *SE* = 0.011, *t*(2,176) = 1.53, *p* = 0.13] compared to neutral lists.

#### Word Identification Speed

As expected, there was a significant effect of grade on the word identification score [*F*(2,110) = 39.61, *p* < 0.001, $\eta_{p}^{2}$ = 0.419]. Planned comparisons demonstrated that the fifth graders’ scores (*M* = 60, *SD* = 12) were lower than the seventh [*M* = 83, *SD* = 15; *F*(1,110) = 36.58, *p* < 0.001] and ninth graders’ scores [*M* = 93, *SD* = 23; *F*(1,110) = 73.52, *p* < 0.001]. In addition, the ninth graders’ word identification scores were higher than the seventh graders’ [*F*(1,110) = 6.15, *p* < 0.05]. As hypothesized, scores increased more between fifth and seventh grades than between seventh and ninth grades.

In the literal task, the models obtained to assess the impact of the word identification score on fifth graders’, seventh graders’, and ninth graders’ search times^4^, respectively, revealed some significant relationship between adolescents’ reading proficiency and search times in the literal task. Indeed, the word identification score significantly predicted the fifth graders’ [β = -0.0037, *SE* = 0.0017, *F*(1,40) = 5.00, *p* < 0.05] and the ninth graders’ [β = -0.0029, *SE* = 0.0011, *F*(1,33) = 6.97, *p* < 0.05] search times, but not the seventh graders’ [β = 0.0006, *SE* = 0.0016, *F*(1,38) = 0.14, *p* = 0.71]. The fifth and ninth graders displaying higher word identification speed found the target words faster. However, the models obtained for the eye movement data^5^ did not reveal any significant impact of the word identification score on either the number of gazes made on non-target words (all *Fs* < 3.31, all *p*s > 0.07) or their average duration (all *Fs* < 2.03, all *p*s > 0.16).

### Discussion

The literal search task was easy for all participants, as indicated by the very low error rate. By the age of 10, students do not have any problem holding a target word in memory while searching for it in a list of nine items. As expected, participants’ search times decreased from fifth to ninth grade because older students fixated non-target words for less time, but also because they made fewer gazes on non-target words. This result demonstrates that even for such a simple search task, developing readers’ search skills improve throughout adolescence, due in part to their increased ability to ignore irrelevant materials based on parafoveal information.

The results of the reading proficiency test were also in accordance with assumptions. Participants’ word identification speed increased by about 40% between fifth and seventh grade, whereas the 12% increase observed from seventh to ninth grade was barely significant. This pattern corroborates the view that word decoding fluency strongly increases up until sixth grade, and then only improves at a much slower rate (Vellutino et al., 1981; Björklund, 1987; Verhoeven and van Leeuwe, 2008; Verhoeven et al., 2011).

As predicted, performance at the literal search task was only weakly related to adolescents’ word identification level. The individual word identification scores were related to the fifth and ninth graders’ search times, but there were no significant relationships between the participants’ scores and their eye movement data. Indeed, participants do not need to access the lexical representation of non-target words to reject them (Stanovich et al., 1978). For instance, they may eliminate straight away any word that does not begin with the same first letter as the target word, or does not have the same length.

The finding that younger adolescents tend to fixate more non-target words before locating the target in the list was unexpected. Indeed, in previous eye-tracking studies with non-verbal items, the progressive decrease of adolescents’ search times with age was due to a decrease of the average duration, but not the number, of the gazes they made on the search display (Huurneman and Boonstra, 2015; Burggraaf et al., 2018). The data obtained with words suggest that older adolescents become more able to make use of some information in parafoveal/peripheral vision (for instance, the length or typographical shape of words), which would allow them to avoid fixating words that do not resemble the target. When scanning displays of words, they may be able to devote enough attentional resources for efficient parafoveal/peripheral visual processing only when word decoding becomes sufficiently automatic and when the quality of their lexical representations reaches a sufficient level. As stated above, high quality lexical representations include well-specified orthographic, phonological and semantic features that are strongly connected to one another. This should allow older adolescents to reject more easily words bearing no relationship to the target word without fixating them, as soon as they can identify through their peripheral vision any feature of the word that does not match the target word template kept in working memory. In other words, high quality lexical representations would give people more opportunity to eliminate target-irrelevant words without really identifying them. The shorter durations of gazes made on non-target words indicates that older adolescents can make faster decisions about their identity, which may also relate to the better quality of their lexical representations (Perfetti, 2007; Verhoeven and Perfetti, 2011).

As expected, the presence of orthographic distractors increased participants’ search times in all age groups. As in adults, orthographic distractors attracted more gazes and were gazed at for longer durations than other words. The impact of orthographic foils did not vary with grade (see Figure 1 and Table 3), which suggests that the ability to use the orthographic/visual features of words for top-down attention guidance within the display is already maximal in fifth graders. As in adults also, the presence of semantic distractors also increased search times, but this increase could not be related to any significant increase in the number and/or duration of the gazes made on semantic distractors or other non-target words (Léger et al., 2012). As discussed in Dampuré et al. (2014), displaying the words in vertical lists rather than on random displays in which a single word can be fixated at a time restricted the explanatory power of eye movement recordings in Léger et al.’s (2012) study.

Contrary to what was expected, the impact of semantic distractors on search times did not vary according to age groups and thus did not increase with age. This result suggests that even though full adult-like fluency in word decoding is not reached before the age of 12, fifth graders aged 10 to 11 years already have enough mental resources for the processing of word semantic features during visual search (Stanovich, 1980; Perfetti, 1992, 2007; Verhoeven and Perfetti, 2011). As stated by Léger et al. (2012) and Dampuré et al. (2014), words bearing a semantic relationship to the target word would attract attention during the search phase because their visual form matches that of words that were pre-activated in memory on learning the target. Hence, the fifth graders’ level of automation of word decoding and quality of lexical representations would already allow them to spot the words that share semantic features with the target word in their peripheral visual field. As stated above, however, the finding that younger adolescents tend to fixate more non-target words than seventh or ninth graders before locating the target in the list suggests that fifth graders are still less able than their older peers to identify as distractors the words that do not resemble the target word without gazing at them directly.

## Categorical Search Task (Experiment 2)

### Results

#### Error Rates

In the categorical task, participants made 113 target selection errors over 1,368 experimental trials, i.e., a 8.3% error rate. The error rate was 8.9% in fifth graders, 9.3% in seventh graders and 6.5% in ninth graders. Non-parametric Kruskal–Wallis ANOVAs revealed no significant effects of grade on the number of errors made when searching through neutral [*H* (2, *N* = 114) = 1.50, *p* = 0.47], orthographic [*H* (2, *N =* 114) = 2.63, *p* = 0.27] or semantic lists [*H* (2, *N* = 114) = 0.13, *p* = 0.94]. However, when collapsing across grades, a non-parametric Friedman’s ANOVA revealed different error rates depending on the type of list [χ^2^*ANOVA* (*N* = 114, *df* = 2) = 8.35, *p* < 0.05]. Wilcoxon signed-rank tests demonstrated that error rates were higher for semantic lists (12.1%) compared to neutral [5.3%; *Z* (*N* = 56, *df* = 1) = 2.81, *p* < 0.01] or orthographic lists [7.5%; *Z* (*N* = 41, *df* = 1) = 2.34, *p* < 0.05]. Neutral and orthographic lists did not produce significantly different error rates [*Z* (*N* = 44, *df* = 1) = 1.12, *p* = 0.26]. Most of the errors made in semantic lists (83.6%) involved the searcher clicking on a semantic distractor instead of the target word. Similarly, most of the errors made in orthographic lists (70.6%) involved the searcher clicking on one orthographic distractor.

#### Search Times

The lower panel of Table 3 displays the adolescents’ search times as a function of grade and type of list. The model for search times^6^ revealed a main effect for grade [*F*(2,102) = 11.60, *p* < 0.001], but the main effect for type of list [*F*(2,152) = 0.47, *p* = 0.63] and the interaction between the two factors [*F*(4,151) = 1.38, *p* = 0.24] were not significant. As expected, the speed of visual search for words increased with age since, regardless of the type of list, fifth graders’ search times were longer than seventh graders’ [β = -0.119, *SE* = 0.040, *F*(1,102) = 10.29, *p* < 0.001] and ninth graders’ [β = -0.176, *SE* = 0.037, *F*(1,111) = 22.43, *p* < 0.001]. However, ninth graders’ search times were not significantly shorter than seventh graders’ [β = -0.072, *SE* = 0.042, *F*(1,99) = 1.98, *p* = 0.16].

#### Eye Movement Data: Numbers of Gazes

**FIGURE 3:**
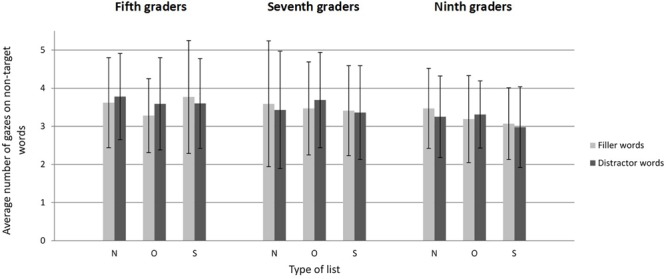
Average number of gazes as a function of grade, type of list, and type of word in Experiment 2 (categorical search). Error bars represent standard deviations. N, Neutral; O, Orthographic; S, Semantic.

Figure 3 displays the average number of gazes made by participants on non-target words as a function of grade, type of list, and type of word. Adolescents gazed at six to seven of the eight non-target words in each list. In contrast to the literal task, they did not always stop searching as soon as they fixated the target word, but sometimes continued exploring the rest of the display.

Like the model for search times, the model for the number of gazes^7^ revealed only a main effect for grade [*F*(2,110) = 3.13, *p* < 0.05]. The remaining main effects and interactions were not significant (all *F*s < 1.06, all *p*s > 0.35). The main effect of grade revealed that part of the increase of search speed with age was due to a decrease of the number of gazes made on non-target words. Indeed, regardless of the type of list and type of word, fifth graders gazed at more non-target words than ninth graders [β = -0.019, *SE* = 0.008, *F*(1,111) = 6.29, *p* < 0.05] but did not gaze at significantly more non-target words than seventh graders [β = -0.010, *SE* = 0.009, *F*(1,113) = 2.19, *p* = 0.14]. Seventh graders did not gaze at significantly more non-target words than ninth graders [β = -0.010, *SE* = 0.009, *F*(1,110) = 1.04, *p* = 0.31].

#### Eye Movement Data: Average Gaze Durations

**FIGURE 4:**
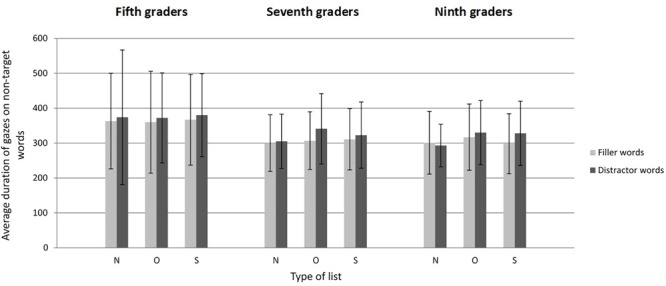
Average duration of gazes as a function of grade, type of list, and type of word in Experiment 2 (categorical search). Error bars represent standard deviations. N, Neutral; O, Orthographic; S, Semantic.

Figure 4 displays mean gaze durations as a function of type of grade, type of list, and type of word. The model for the average duration of gazes^8^ revealed only a marginally significant main effect for grade [*F*(2,111) = 2.73, *p* = 0.07], which suggests that the increase of search speed with age did not mostly result from a decrease of the duration of gazes made on non-target words. However, there were main effects for type of list [*F*(2,99) = 3.40, *p* < 0.05] and type of word [*F*(1,822) = 4.88, *p* < 0.05], and an interaction between type of list and type of word [*F*(2,2.043) = 2.94, *p* = 0.05]. The other interactions were not significant (all *F*s < 1.51, all *p*s > 0.19).

As hypothesized, the follow-up pairwise comparisons associated with the type of list by type of word interaction demonstrated that regardless of grade, participants gazed longer at orthographic distractors than at neutral distractors [β = 0.040, *SE* = 0.013, *t*(326) = 3.05, *p* < 0.01], and also longer at semantic distractors than at neutral distractors [β = 0.032, *SE* = 0.013, *t*(298) = 2.52, *p* < 0.05]. Adolescents displayed similar gaze durations on filler words in orthographic lists [β = -0.002, *SE* = 0.012, *t*(332) = 0.10, *p* = 0.92] and semantic lists [β = 0.013, *SE* = 0.013, *t*(303) = 0.94, *p* = 0.35] compared to neutral lists.

#### Word Identification Speed

In the categorical task, the models obtained to assess the impact of the word identification score on adolescents’ search times^9^ revealed that the word identification score strongly predicted both fifth graders’ [β = -0.0176, *SE* = 0.0034, *F*(1,38) = 26.32, *p* < 0.001] and ninth graders’ search time [β = -0.0106, *SE* = 0.0019, *F*(1,33) = 31.02, *p* < 0.001]. As expected, the students displaying higher word identification speed found the target words faster. The same was true for seventh graders, but the relationship between the students’ word identification score and search time did not reach significance [β = -0.0051, *SE* = 0.0032, *F*(1,27) = 2.50, *p* = 0.12].

The models obtained to assess the impact of the word identification score on the number of gazes made on non-target words^10^ did not reveal any significant impact of the word identification score (all *F*s < 3.18, all *p*s > 0.08) on this dependent variable. In contrast, the models obtained for the average duration of these gazes^11^ revealed that the word identification score predicted their average duration for fifth graders’ [β = -0.0048, *SE* = 0.0015, *F*(1,41) = 9.74, *p* < 0.01] and ninth graders’ [β = -0.0033, *SE* = 0.0005, *F*(1,35) = 40.49, *p* < 0.001]. The students displaying higher word identification speed gazed for shorter times at non-target words. The same was true for seventh graders, but the relationship between the students’ word identification score and the average duration of gazes did not reach significance [β = -0.0016, *SE* = 0.0011, *F*(1,29) = 2.03, *p* = 0.16]. Hence, the relationship between adolescents’ word identification scores and search times was underpinned by an impact of the word identification score on the duration of gazes made on non-target words. Altogether, performance and eye movement data were more strongly related to adolescents’ word identification levels in the categorical than in the literal task.

### Discussion

Comparison with the literal task (Table 3) shows that search times were two to three times longer in the categorical task. The number of gazes made while exploring the display increased to almost one gaze per word (compare Figure 1, 3). Average gaze durations were also longer (compare Figure 2, 4), which reflects the deeper processing required when the target word is only defined by a semantic cue. Even if there were more errors in the categorical task compared to the literal task, the error rate was low (less than 10%) and did not significantly vary with age. As in the literal task, participants could apparently manage to keep the categorical cue in working memory while exploring the display and assessing the non-target words. Most of the errors consisted in clicking on either an orthographic neighbor or a semantic associate of the target word and were, thus, probably due to false alarms and speedy decisions.

As in the literal task, participants’ search times decreased from fifth to ninth grade. This decrease was due mostly to a progressive diminution of the number of gazes made on non-target words, but was also linked to a shortening of the average duration of gazes that was, however, only marginally significant, probably because of the large variability of fifth graders’ gaze durations (see Figure 4). The shortening of gaze durations observed between fifth and seventh grade may reflect the acquisition of fluent word decoding abilities by sixth grade (Vellutino et al., 1981; Verhoeven and van Leeuwe, 2008; Verhoeven et al., 2011). The reduction of the number of gazes may correspond to an increased ability to process word features in the parafoveal/peripheral visual field (Perfetti, 2007; Siéroff and Riva, 2011; Verhoeven and Perfetti, 2011), as already suggested for the literal task (see discussion of Experiment 1).

As expected, there was a strong negative relationship between the fifth graders’ and ninth graders’ word identification score and their search rates in the categorical task, such that better readers took less time to find the target words compared to poorer readers. The same trend was seen in seventh graders. These faster search times were due to a decrease in the duration of better readers’ gazes on non-target words. Indeed, since in this task the target word is not known in advance, but only defined by a categorical cue, participants must access the meaning of each word they gaze at before they are able to tell whether it is the target word or not.

Studies conducted with adult participants (Léger et al., 2012) showed that semantic distractors induced a higher rate of target selection errors in the categorical task, but did not significantly increase search times or the number or duration of gazes made while searching. In the present work, the impact of semantic distractors on gaze durations and search times was expected to be stronger for adolescents than for adults, and stronger in the categorical than in the literal search task. The first hypothesis was verified since whatever their grade, and in contrast with adult participants (Léger et al., 2012), adolescents gazed longer at semantic distractors than at neutral distractors in the categorical task. The second hypothesis was also verified since as reported above (see Experiment 1), adolescents did not gaze significantly more often or longer at semantic distractors than at neutral words in the literal task. Finally, similar to adults, adolescents performing the categorical task made more target selection errors in semantic than in orthographic or neutral lists.

A last hypothesis was that the impact of orthographic distractors on gaze durations and search times was expected to increase with age, because it involves a pre-activation of potential target words that is mediated by semantic features, i.e., by automatic spreading activation within the semantic network. As such, as stated in the Introduction, the impact of orthographic distractors in the categorical task was expected to increase with age just like the impact of semantic distractors in the literal task, due to a more automatic activation of words’ semantic features in older adolescents (Perfetti, 2007; Verhoeven and Perfetti, 2011). However, the impact of orthographic distractors did not increase with age since, whatever their grade, adolescents made longer gazes on orthographic distractors than on neutral words. These data are consistent with the fact that, as reported above, the impact of semantic distractors did not increase with age either in the literal task, and corroborate the idea that the impact of orthographic distractors in the categorical task and the impact of semantic distractors in the literal task result from the same spreading activation mechanism within the semantic network.

## General Discussion

### Development of Visual Search for Words During Adolescence

These two experiments aimed at identifying patterns of development in children and teenagers’ scanning of word displays. Altogether, the data demonstrate that the efficiency of visual search for a single word within other words steadily increases with age in adolescents between the ages of 10 to 16.

Progress in visual search efficiency was associated with a strong increase in word identification skills, which were a strong determinant of average gaze durations and search times for the categorical task, but much less for the literal task. Visual search for words became more efficient in both the literal and categorical search tasks both because older adolescents gazed less often at non-target words during the search and because they could reject non-target words more expeditiously once they were fixated. This supports the assumption that the progressive automation of word decoding facilitates the rejection of non-target words and makes more mental resources available for parafoveal/peripheral word processing (Siéroff and Riva, 2011) and top-down guidance within word lists. In addition, the lexical quality hypothesis (Perfetti, 1992, 2007; Verhoeven and Perfetti, 2011; Verhoeven et al., 2011) states that the acquisition of word decoding is accompanied by an experience-related increase in the lexical quality of word representations within semantic memory, which would go on during the whole adolescence and through to adulthood. The better quality of older adolescents’ word representations would facilitate a flexible use of both the perceptual and semantic features of words for top-down guidance within the displays, and increase the likelihood that they select potential target words and reject irrelevant words without fully identifying them and gazing at them directly.

In the literal search task, orthographic distractors increased adolescents’ search times and number of gazes on non-target words in all age groups, as in adults (Léger et al., 2012; Dampuré et al., 2014). Contrary to what was expected, the impact of semantic distractors on search times did not increase with age, which means that participants of all grades were equally vulnerable to semantic distractors. This suggests that despite their lower reading abilities and the lower quality of their lexical representations, fifth graders performing the literal search task can already spot in their peripheral visual field, as well as ninth graders and adults, the words that share either orthographic/visual or semantic features with the target word.

In the categorical search task, interestingly, the impact of semantic distractors on gaze durations was stronger for all adolescents than for the adults tested by Léger et al. (2012), which suggests that ninth graders’ resistance to semantic interference is not as strong yet as that of adults. According to the literature on executive functions development (Brocki and Bohlin, 2004; Gathercole et al., 2004; Yurgelun-Todd, 2007), part of the progressive increase in adolescents’ word search abilities may also result from the concomitant maturation of executive functions, namely the inhibitory processes and their interaction with working memory and attention control.

### Implications of the Current Work for Adolescents’ Visual Search in Internet-Like Environments

Data from the present experiments show that the development of abilities to quickly spot and select a target word within word lists between the 5th and the 9th grades is strikingly similar to the development of information search abilities within more complex environments such as websites or pages (Hirsh, 2000; Wallace et al., 2000; Rouet et al., 2011). However, the literature reporting adolescents’ behavior in more complex reading tasks involving the scanning and detection of relevant words, such as searching from search engine result pages (Rouet et al., 2011; Keil and Kominsky, 2013), suggested that the impact of semantic distractors on visual search for words should increase with age. This particular hypothesis was not verified because in the literal search task, fifth graders were already able to spot the words that shared semantic features with the target word in the search field as well as ninth graders or adults. This suggests that because the literal search task is much easier to perform than complex tasks involving the scanning and detection of relevant words such as Web search, it is not sensitive enough to reveal fifth graders’ difficulties in selecting semantically relevant phrases in response to a search phrase observed in real-life situations. Additional experiments involving younger children such as third or fourth graders should be conducted to check whether, as may be expected, the impact of semantic distractors on their performance in the literal search task is lower compared to the adolescents tested in this study.

The present data may have several instructional implications for optimizing adolescents’ verbal information search. Firsmilar progression of the matutly, when the target word is known, the efficiency of the search is relatively independent from adolescents’ word identification speed. This may explain why many young adolescents favor keyword search strategies while seeking information (Walraven et al., 2008; Zhang and Quintana, 2012). Indeed, even poor or average readers will be able to perform the task with apparent success as long as they must only locate predefined keywords, which might actually have fueled the common illusion that children and adolescents master the Internet much better than adults do. Obviously, to consider the context and eliminate irrelevant hits is much more demanding and will use the pupils’ reading and word decoding abilities.

Secondly, the overall similar progression of the maturation of single word visual search processes and that of more real-life information search within complex verbal documents suggests that young adolescents’ difficulties in performing fruitful information search on the web are linked to the progressive improvement of adolescents’ lexical representations and word decoding abilities during that period. As such, these factors may be difficult to overcome if the students are not properly guided while performing such kind of work. A rather simple way of preparing adolescents to search for information on the web would be to enrich their knowledge of the topic of the search and of the related vocabulary. For instance, Rouet et al. (2011, Experiment 2) found that fifth graders were actually able to consider the meaning of websites titles better and eliminate irrelevant search results if they had previously read a small text elaborating on the search topic. Hence, they would be able to take into account word meanings better during web-related information searches when they have a richer representation of the topic, i.e., when they had the occasion to enrich their topic-related vocabulary.

Thirdly, the data support that, when the exact wording of the information to find is unknown (a frequent event in real life), word identification speed as well as the extent of adolescents’ vocabulary all become strong determinants of search success. In this more difficult situation, the quality of adolescents and adults’ lexical representations should be of utmost importance. Since the lexical quality variations that arise through general literacy and language experiences such as practice in reading and writing are also essential for text comprehension (Perfetti, 2007), the ultimate determinants of the success of adolescents’ complex information search should be similar to those of more “classical” reading and comprehension abilities.

Of course, one limitation of the current work is that in real life, people do not always search for words through well-arranged lists of information, which questions the generalizability of the findings to other contexts. Particularly given the proliferation of different website designs, information is often scattered across the screen with no immediate interpretable way to locate the information one seeks. As such, the current restriction to situations of list searches could spur future research on visual search through more random displays. Finally, the current work is limited in the sense that we have not directly tested the quality of lexical representations *per se*.

## Ethics Statement

This study was carried out with written informed consent from all subjects in accordance with the Declaration of Helsinki. The experiments were conducted in two elementary schools and two junior high schools. Permission was sought from the school, the teacher, and from all students’ parents by sending a parental consent form. After permission was obtained, all students were read a verbal script either individually or during a whole-class session and were asked to participate in the study. All of the children who accepted to participate were included. At the time the study was started (fall of 2010) behavioral research with typical participants was not subjected to prior ethical reviewing in France, therefore no Institutional Review Board approval was required.

## Author Contributions

NV, JB, CR, and J-FR conceived and designed the study and the experimental material. NV, JB, DD, and CR conducted the experiments and analyzed the data. JB and NV wrote the first draft of the manuscript. AP and J-FR made substantial contributions to the interpretation of the data and wrote sections of the manuscript. NJ revised the manuscript critically for important intellectual content. All authors contributed to manuscript revision, read, and approved the submitted version.

## Conflict of Interest Statement

The authors declare that the research was conducted in the absence of any commercial or financial relationships that could be construed as a potential conflict of interest.

## Footnotes

1 logST ∼ grade ^∗^ type of list + (1 | participants) + (1 + type of list + grade | item).

2 logNG ∼ grade ^∗^ type of word ^∗^ type of list + (1 | participants) + (1 + type of word + type of list | item).

3 logADG ∼ grade ^∗^ type of word ^∗^ type of list + (1 + type of word + type of list | participants) + (1 + type of word + type of list | item).

4 logST ∼ word identification score + type of list + (1 | participants) + (1 | item).

5 logNG ∼ word identification score + type of word ^∗^ type of list + (1 | participants) + (1 | item); logADG ∼ word identification score + type of word ^∗^ type of list + (1 + type of word | participants) + (1 + type of word | item).

6 ST ∼ grade ^∗^ type of list + (1 + type of list | participants) + (1 + grade | item).

7 logNG ∼ grade ^∗^ type of word ^∗^ type of list + (1 + type of list | participants) + (1 + type of word + type of list | item).

8 logADG ∼ grade ^∗^ type of word ^∗^ type of list + (1 + type of list | participants) + (1 + type of word | item).

9 logST ∼ word identification score + type of list + (1 + type of list | participants) + (1 + type of list | item).

10 logNG ∼ word identification score + type of word ^∗^ type of list + (1 | participants) + (1 + type of word | item).

11 logADG ∼ word identification score + type of word ^∗^ type of list + (1+ type of word + type of list | participants) + (1 + type of word | item).
